# Apolipoprotein E and Alzheimer's disease pathology in a diverse autopsy study

**DOI:** 10.1002/alz.70927

**Published:** 2025-11-21

**Authors:** Claudia K. Suemoto, Regina Paradela, Renata E. P. Leite, Vitor R. Paes, Carlos A. Pasqualucci, Eduardo Ferriolli, Mayana Zatz, Gabriel do Nascimento Santos, Marvin Afonso Longo Salvador de Alexandria, Ana C. Pereira, Juan Fortea, Michel Naslavsky, Lea T. Grinberg

**Affiliations:** ^1^ Division of Geriatrics Faculdade de Medicina Universidade de Sao Paulo Sao Paulo Brazil; ^2^ Department of Pathology Faculdade de Medicina Universidade de Sao Paulo Sao Paulo Brazil; ^3^ Institute of Biosciences Universidade de Sao Paulo Sao Paulo Brazil; ^4^ Department of Neurology Icahn School of Medicine at Mount Sinai New York New York USA; ^5^ Nash Family Department of Neuroscience Friedman Brain Institute Icahn School of Medicine at Mount Sinai New York New York USA; ^6^ Sant Pau Memory Unit Neurology Department Institut d'Investigacions Biomèdiques Sant Pau Hospital de la Santa Creu i Sant Pau Universitat Autònoma de Barcelona Barcelona Spain; ^7^ Departments of Laboratory Medicine and Pathology and Neurosciences Mayo Clinics Jacksonville Florida USA

**Keywords:** Alzheimer's disease, apolipoprotein, genetics, race, risk factors

## Abstract

**INTRODUCTION:**

Apolipoprotein E allele 4 (APOE ε4) is the main genetic risk factor for late‐onset Alzheimer's disease (AD), but most evidence comes from White populations in high‐income countries. We investigated APOE and AD pathology in an ethnically diverse Brazilian autopsy cohort.

**METHODS:**

This cross‐sectional study used Biobank for Aging Studies (BAS) data. AD pathology was evaluated with Braak, Consortium to Establish a Registry for Alzheimer's Disease, and Thal criteria. APOE genotypes were obtained from blood or brain tissue. Regression models were adjusted for age, sex, race, and education; interaction with race was tested.

**RESULTS:**

Among 1391 participants (mean age 75.1 ± 12.4 years, 50% women, 64% White), APOE ε4showed a dose response with greater AD pathological burden and higher odds of AD diagnosis, while APOE ε2/X was protective. APOE ε4/ε4 did not show full penetrance across age groups. Associations were similar in White and Black individuals.

**DISCUSSION:**

APOE ε4was strongly associated with AD pathology, with consistent associations across racial groups.

**Highlights:**

- APOE ε4 increased AD neuropathological burden in Brazilians.
- APOE ε2/εX was protective against AD pathology.
- APOE ε4/ε4did not show full penetrance across age groups.
- Associations between APOE and AD pathology were similar by race.
- Large multiethnic autopsy study expands evidence beyond high‐income country studies.

## 1 BACKGROUND

Dementia is a major global health challenge, affecting approximately 57 million people worldwide in 2019, with projections indicating an increase to 153 million by 2050.^1^ This rising prevalence poses significant societal and economic burdens, with estimated global costs reaching US$ 1.3 trillion in 2019.^2^ In addition to financial costs, dementia places a heavy strain on caregivers, who often experience emotional, physical, and economic hardships.^3^ Among the various causes of dementia, Alzheimer's disease (AD) is the most common, accounting for 50% to 80% of cases, alone or together with other neuropathologies.^4^, ^5^ Although AD is influenced by multiple genetic and environmental factors, genetic predisposition plays a crucial role, with the apolipoprotein E epsilon 4 (APOE ε4) allele being the strongest known genetic risk factor for late‐onset AD.^6^, ^7^

The association between APOE ε4 and AD has been widely studied, with recent findings suggesting variability in the penetrance to AD pathology across different populations. A study by Fortea et al. using data from the National Alzheimer's Coordinating Center (NACC) reported near‐full penetrance of APOE ε4 in homozygous individuals, meaning that almost all individuals carrying two copies of the allele would develop AD pathology if they reached a certain age.^8^ However, other studies have found varying degrees of penetrance, suggesting that additional genetic and environmental factors influence disease development. Indeed, while APOE ε4 represents the strongest known genetic risk factor for AD, a recent review emphasized that genetics alone does not fully explain disease variance, with estimates suggesting that approximately 60% of AD risk remains unexplained by genetics.^9^ Moreover, reports underscore the crucial role of modifiable risk factors, such as cardiovascular health, education, and lifestyle, in shaping dementia risk, particularly in low‐ and middle‐income countries.^10^, ^11^ Taken together, these findings point to a complex interplay between genetic and environmental factors in determining AD risk.

Notably, the association between APOE ε4 and the clinical diagnosis of AD‐type dementia appears to differ by race, with some studies reporting a stronger association in White individuals compared to Black individuals.^12^, ^13^ These disparities suggest the need for more diverse studies to investigate the penetrance of APOE ε4 homozygosity in causing AD pathology using clinicopathological cohorts. While autopsy studies remain the gold standard for confirming AD diagnosis and distinguishing it from other causes of dementia, most previous autopsy studies were heavily enriched for White individuals from the United States (US), limiting the generalizability of their findings to other populations.^8^, ^14^, ^15^ To better understand APOE penetrance, we aimed to determine the association between APOE genotypes and AD pathology in a large, ethnically diverse autopsy study in Brazil.

## 2 METHODS

### 2.1 Participants

In this cross‐sectional study, we used data from the Biobank for Aging Studies (BAS) from the University of Sao Paulo Medical School collected between 2004 and 2024.^4^ The BAS gathered clinical data and biological material in the Sao Paulo Autopsy Service, where full‐body autopsies were performed to determine the cause of death in people who died in the city of Sao Paulo (Brazil) from natural deaths with unclear etiology. BAS inclusion criteria were age 18 years or older, the presence of an informant who had at least weekly contact with the deceased 6 months before death and could provide reliable clinical information, and signed informed consent. The exclusion criteria were cerebrospinal fluid pH < 6.5 or major brain lesions (e.g., hemorrhages, large infarcts, tumors) that required brain retention by the pathologist to determine the death cause. For this study, we excluded cases with incomplete data for any variable of interest (Figure S1).

### 2.2 Clinical evaluation

Age and sex information were collected from government‐issued documents. Other sociodemographic and clinical data were collected with an informant by trained gerontologists in a private room using a structured interview.^16^ Race was reported by the informant following the Brazilian Census categories based on skin color: Black, Brown (mix of Black and White race), White, and Yellow (i.e., Asian). We combined the Black and Brown groups into one category referred to as Black due to similar socioeconomic and racism burdens.^17^

Cognitive abilities were evaluated using the Clinical Dementia Rating (CDR).^18^ By study design, only the informant part was applied. Participants were classified as having normal cognition (CDR = 0), questionable dementia (CDR = 0.5), or dementia (mild: CDR = 1, moderate: CDR = 2, or severe: CDR = 3).

### 2.3 Neuropathological evaluation

Brains were collected within 24 h after death. One hemisphere was fixed in 4% buffered paraformaldehyde for 14 days, and the other was frozen at −80°C for future studies. We sampled the following areas from the fixed hemisphere: middle frontal gyrus, middle and superior temporal gyri, angular gyrus, superior frontal and anterior cingulate gyrus, visual cortex, hippocampal formation at the level of the lateral geniculate body, amygdala, basal ganglia at the level of the anterior commissure, thalamus, midbrain, pons, medulla oblongata, and cerebellum. These areas were blocked in paraffin and cut into 5‐µm‐thick sections. AD pathology was identified using immunohistochemistry with antibodies against Aβ (4G8, 1:10.000; BioLegend Catalogue No. 800701) for the Consortium to Establish a Registry for Alzheimer's Disease (CERAD) and Thal classifications^19^, ^20^ and phosphorylated tau (AT8, 1:400; Invitrogen MN1020) for neurofibrillary tangles using the Braak and Braak staging system.^21^

**RESEARCH IN CONTEXT**

1. **Systematic review**: We searched PubMed for autopsy studies published up to 2024 that examined the association between APOE genotypes and AD neuropathology. Most studies were conducted in White populations from high‐income countries, with limited data from diverse or low‐ and middle‐income settings.
2. **Interpretation**: In a large multiethnic autopsy cohort from Brazil, APOE ε4 was associated with greater AD pathological burden and increased odds of AD neuropathological diagnosis, while APOE ε2/εX was protective. Contrary to prior assumptions, APOE ε4/ε4 did not show full penetrance across age groups, and associations were consistent across racial groups. These findings extend previous work by demonstrating the robustness of APOE effects beyond high‐income, predominantly White populations.
3. **Future directions**: Future research should investigate gene–environment interactions, assess longitudinal clinical–pathological correlations in diverse populations, and explore biological mechanisms underlying incomplete penetrance of APOE ε4/ε4.

The CERAD staging determines the frequency of neuritic plaques using a semiquantitative approach into four stages: none (0), sparse (A), moderate (B), and frequent (C).^19^ The Thal classification has five phases.^20^ Aβ deposits are found only in the neocortex in the first phase. Phase 2 is characterized by the involvement of the allocortical brain regions. In phase 3, diencephalic nuclei, the striatum, and the cholinergic nuclei of the basal forebrain have Aβ deposits. Other brainstem nuclei become involved in phase 4, while phase 5 is characterized by cerebellar deposition.

The Braak staging is a system to measure the progression of neurofibrillary tangles (NFTs) into six stages. Stages I and II are characterized by NFT deposition in the transentorhinal and entorhinal cortex. As the pathology progresses, the lesions extend to limbic regions (stages III and IV). Extensive neocortical NFT involvement is present at stages V and VI. AD diagnosis was ascertained in cases with intermediate or high AD neuropathological changes (e.g., CERAD was B or C and the Braak stage was III or more). For the analysis, Braak and Braak staging, CERAD scores, and Thal phases were grouped into none, low, intermediate, and high, also following NIA‐Alzheimer's Association Criteria.^22^

### 2.4 APOE genotyping

DNA samples were obtained from blood or brain tissue from cerebellum samples collected within 24 h after death and genotyped using Illumina OmniExpress 700k microarray or Illumina BeadXpress custom genotyping panel.^13^ APOE alleles were genotyped directly using allele‐specific amplification.^23^ For a small proportion of cases (*n* = 116), APOE alleles were determined after imputation of rs429358 to compose haplotypes.^24^ Individuals were classified into four groups: APOE ε2/εX (which includes APOE ε2/ε2 and APOE ε2/ε3), APOE ε3/ε3, APOE ε3/ε4, and APOE ε4/ε4. Individuals with APOE ε2/ε4 were excluded because of the opposite associations of APOE ε2 and APOE ε4 on AD risk and the small sample size of this group.^15^

### 2.5 Statistical analysis

We used descriptive statistics to compare the demographic and clinical characteristics of the sample across the APOE genotyping groups. We employed the chi‐squared or Fisher tests for categorical variables and ANOVA or Kruskal–Wallis tests for continuous variables. To investigate the association between age and neuropathological markers by APOE genotypes, we plotted the distribution of Braak, CERAD, and Thal staging and AD neuropathological diagnosis across different age groups (50s, 60s, 70s, 80s, and 90s), races (White vs Black), and informant‐based cognitive function levels based on the CDR score (normal cognition: CDR = 0, questionable dementia: CDR = 0.5, and dementia: CDR ≥ 1). These distributions were examined within each APOE genotype group (APOE ε4/ε4, APOE ε3/ε4, APOE ε3/ε3, and APOE ε2/εX). This approach enabled us to visually identify patterns of penetrance and potential differences in neuropathological burden associated with APOE genotypes according to sociodemographic and informant‐based cognitive status. We also plotted the frequency of AD and non‐AD/normal neuropathological diagnoses according to APOE genotypes and stratified by informant‐based cognitive status (CDR = 0, 0.5, and ≥ 1) and race (White vs Black). The mean age of death with 95% confidence intervals was calculated by APOE genotypes and compared using one‐way ANOVA for the overall sample and in participants with AD pathology stratified by informant‐based cognitive status and race.

To assess the association of APOE genotypes with AD pathology (defined as Braak staging ≥ 3 and a CERAD score ≥ 2) and neuropathological markers, we applied ordinal or binary logistic regression models adjusted for age, sex, race, and education. These analyses were conducted on the whole sample, as well as only on participants with dementia and stratified by race. Additionally, we examined whether the association between APOE genotypes and AD pathology was modified by race by including interaction terms between APOE and race in the regression models. Statistical analyses were performed using R version 4.4.1, with an alpha level set at 0.05.

## 3 RESULTS

### 3.1 Sample characteristics

We included 1391 participants with a mean age of 75.1 (12.4) years old, 50% were women, 64% were White, and the mean education was 5.1 (4.2) years (Table 1). Twenty‐nine percent of the sample had at least one APOE ε4 allele, but the frequency of APOE ε4/ε4 was only 3%. Participants with the APOE ε4/ε4 genotype had lower educational levels than other groups. Additionally, the distribution of APOE groups differed by race, with Black participants showing a relatively higher frequency of APOE ε2/εX and APOE ε3/ε4 compared to White participants. As expected, APOE ε4/ε4 was associated with advanced Braak, Thal, and CERAD stages, as well as with dementia (CDR ≥ 1) (Table 1).

**TABLE 1:** Demographic and clinical characteristics of the sample by APOE genotypes (*n* = 1391).

Variables	Overall (*n* = 1391)	APOE ε2/X (*n* = 135)	APOE ε3/ ε3 (*n* = 852)	APOE ε3/ ε4 (*n* = 361)	APOE ε4/ ε4 (*n* = 43)	*p*
Age at death, mean (SD) years^a^	75.1 (12.4)	74.1 (12.6)	75.1 (12.8)	75.2 (11.4)	77.3 (12.6)	0.53
Education, mean (SD) years^a^	5.1 (4.2)	5.2 (4.1)	5.3 (4.2)	4.9 (4.2)	3.7 (3.7)	0.05
Female, *n* (%)^b^	692 (49.8)	63 (46.7%)	427 (50.1%)	178 (49.3%)	24 (55.8%)	0.75
Race, *n* (%)^b^						<0.001
White	888 (63.8)	73 (54.0)	581 (68.2)	206 (57.1)	28 (65.1)	–
Black	465 (33.4)	58 (43.0)	244 (28.6)	149 (41.3)	14 (32.6)	–
Other	38 (2.8)	4 (3.0)	27 (3.2)	6 (1.6)	1 (2.3)	–
Braak stages, *n* (%)^b^						<0.001
0	218 (15.7)	28 (20.7)	140 (16.4)	49 (13.6)	1 (2.2)	–
I–II	474 (34.0)	55 (40.7)	301 (35.3)	104 (28.8)	14 (32.6)	–
III–IV	485 (34.9)	43 (31.9)	306 (36.0)	126 (34.9)	10 (23.3)	–
V–VI	214 (15.4)	9 (6.7)	105 (12.3)	82 (22.7)	18 (41.9)	–
CERAD score, *n* (%)^b)^						<0.001
None	798 (57.4)	106 (78.5)	545 (64.0)	138 (38.2)	9 (20.9)	–
Sparse	210 (15.1)	16 (11.9)	113 (13.3)	74 (20.6)	7 (16.3)	–
Moderate	205 (14.7)	8 (5.9)	113 (13.3)	72 (19.9)	12 (27.9)	–
Frequent	178 (12.8)	5 (3.7)	81 (9.4)	77 (21.3)	15 (34.9)	–
Thal stages, *n* (%)^b)^						<0.001
0	520 (42.0)	81 (66.9)	360 (47.5)	75 (23.4)	4 (9.8)	
1	205 (16.6)	19 (15.7)	129 (17.0)	52 (16.3)	5 (12.2)	
2	114 (9.2)	9 (7.4)	64 (8.4)	37 (11.6)	4 (9.8)	
3	157 (12.7)	6 (5.0)	81 (10.7)	65 (20.3)	5 (12.2)	
4	201 (16.3)	5 (4.1)	101 (13.3)	78 (24.4)	17 (41.5)	
5	43 (3.5)	1 (0.8)	23 (3.0)	13 (4.1)	6 (14.6)	
AD neuropathological diagnosis, *n* (%)^b^	345 (24.8)	12 (8.9)	176 (20.7)	133 (36.8)	24 (55.8)	<0.001
CDR score, *n* (%)^b^						<0.001
CDR = 0	936 (67.3)	96 (71.1)	599 (70.3)	223 (61.8)	18 (41.9)	–
CDR = 0.5	121 (8.7	6 (4.4)	84 (9.9)	27 (7.5)	4 (9.3	–
CDR ≥ 1	334 (24.0)	33 (24.4)	169 (19.8)	111 (30.7)	21 (48.8)	–

*Note*: APOE ε2/εX represents APOE ε2/ε2 (*n* = 8) and APOE ε2/ε3 (*n* = 127); APOE ε2/ε4 was excluded from the analyses.

Abbreviations: AD, Alzheimer's disease; APOE, apolipoprotein E gene. CDR, Clinical Dementia Rating; CERAD, Consortium to Establish a Registry for Alzheimer's disease; SD, standard deviation.

^a^ One‐way ANOVA.

^b^ Chi‐squared test.

### 3.2 Distribution of AD pathology and mean age of death by APOE genotypes

**FIGURE 1:**
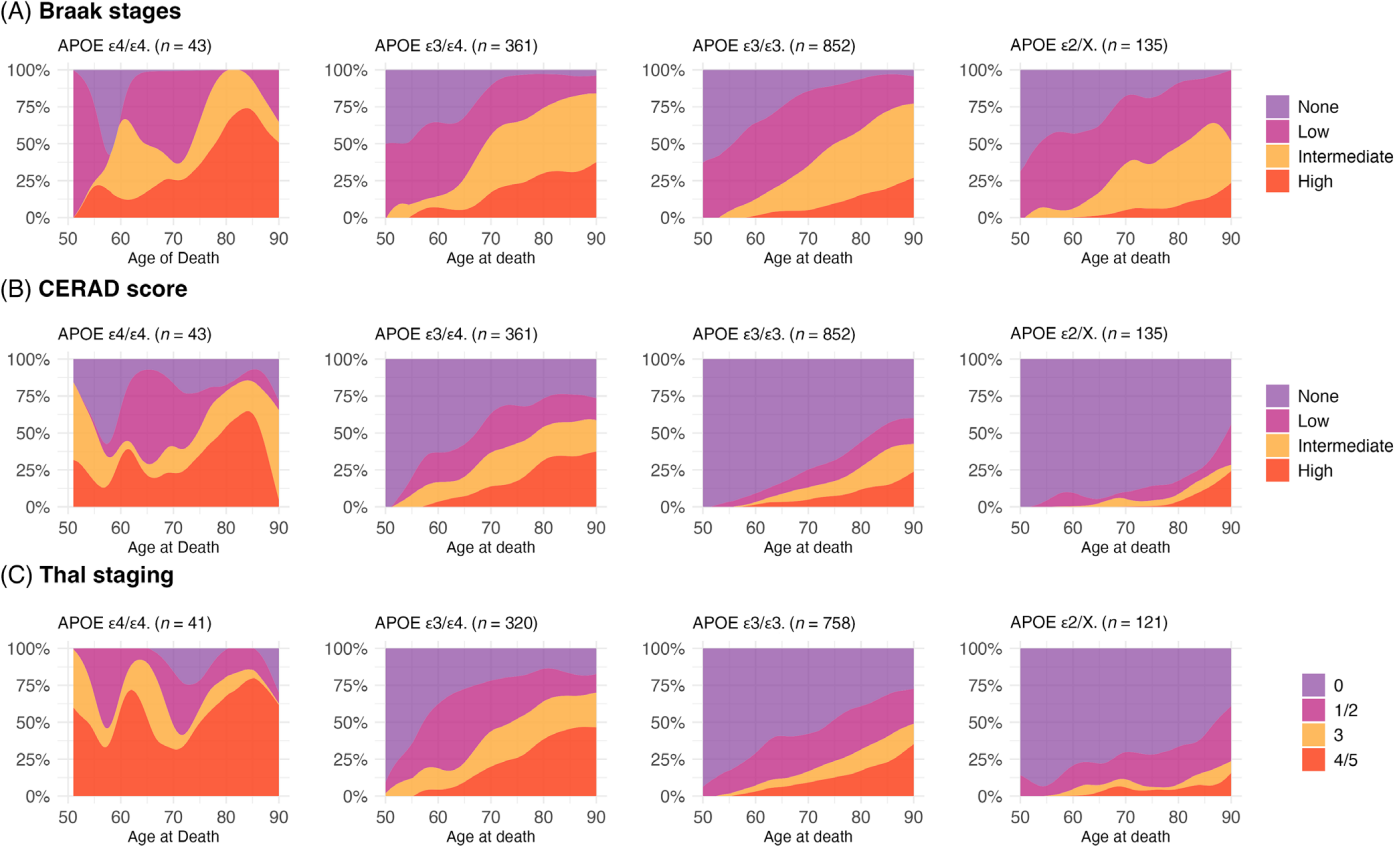
Distribution of (A) Braak stages, (B) CERAD scores, and (C) Thal staging by age of death and APOE genotypes in the whole sample (*n* = 1391). APOE, apolipoprotein E gene; CERAD, Consortium to Establish a Registry for Alzheimer's Disease.

**FIGURE 2:**
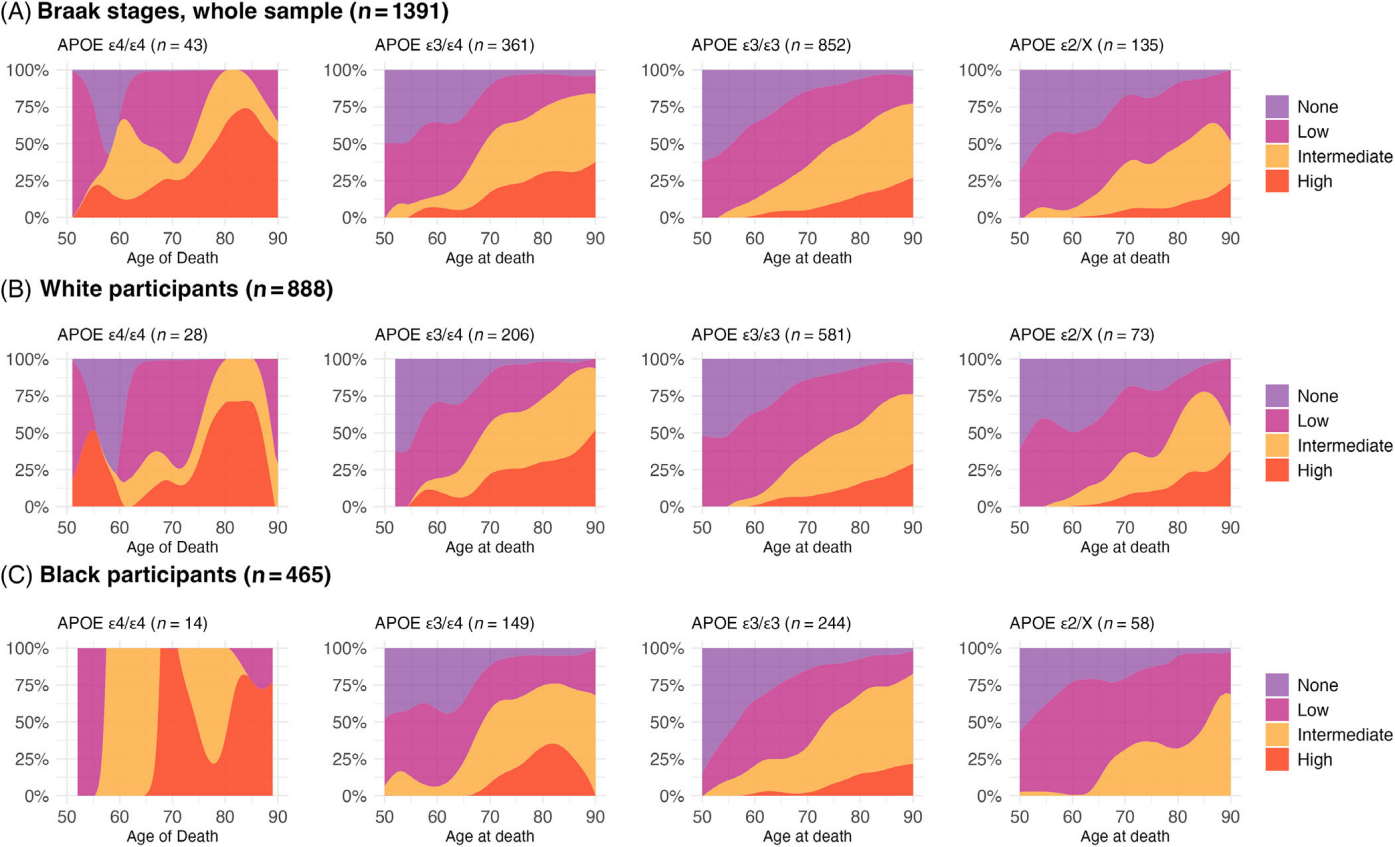
Distribution of Braak stages by age of death and APOE genotypes in the whole sample (A) and stratified by race (B and C). APOE, apolipoprotein E gene.

**FIGURE 3:**
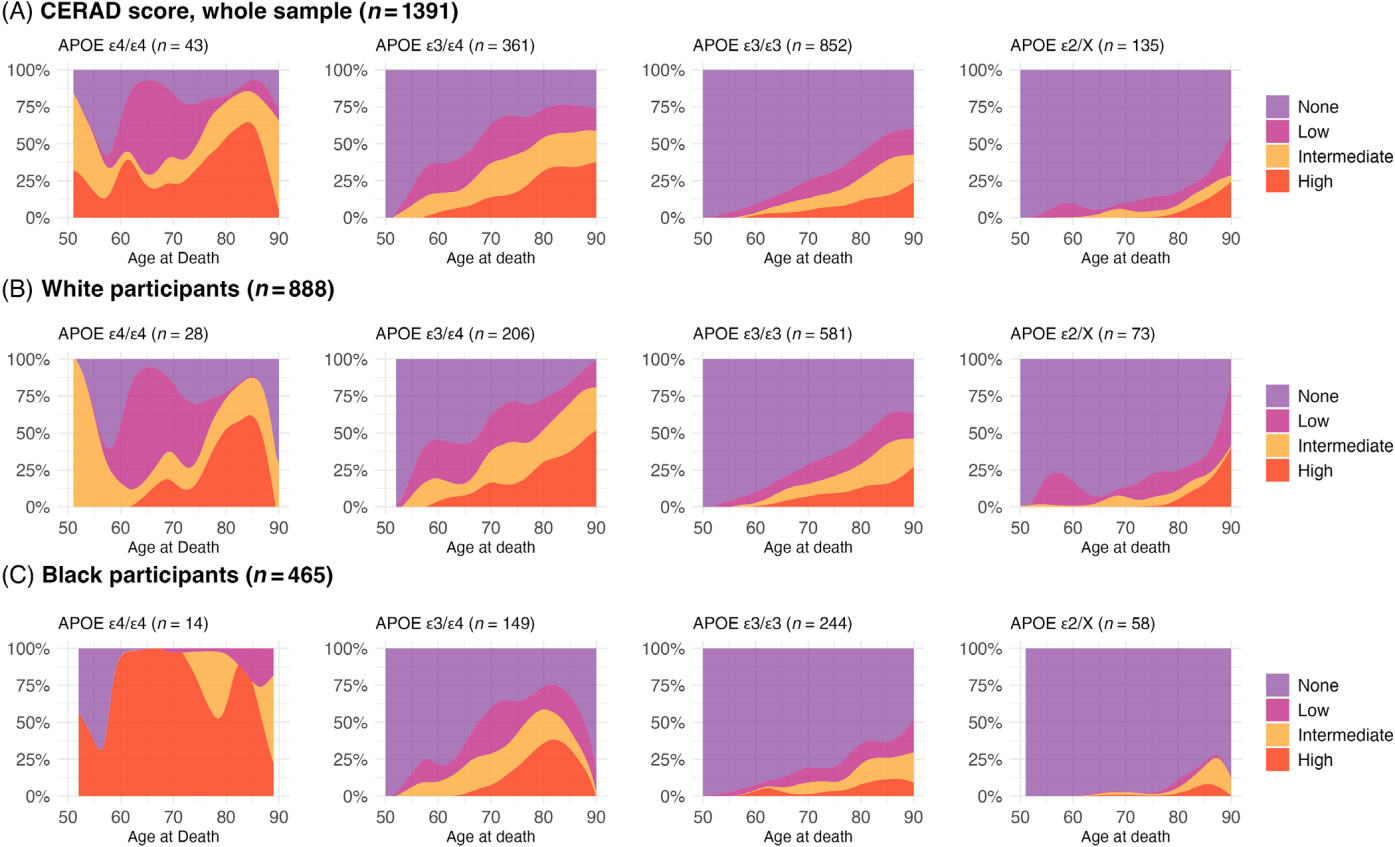
Distribution of CERAD scores by age of death and APOE genotypes in the whole sample (A) and stratified by race (B and C). APOE, Apolipoprotein E gene; CERAD, Consortium to Establish a Registry for Alzheimer's Disease.

**FIGURE 4:**
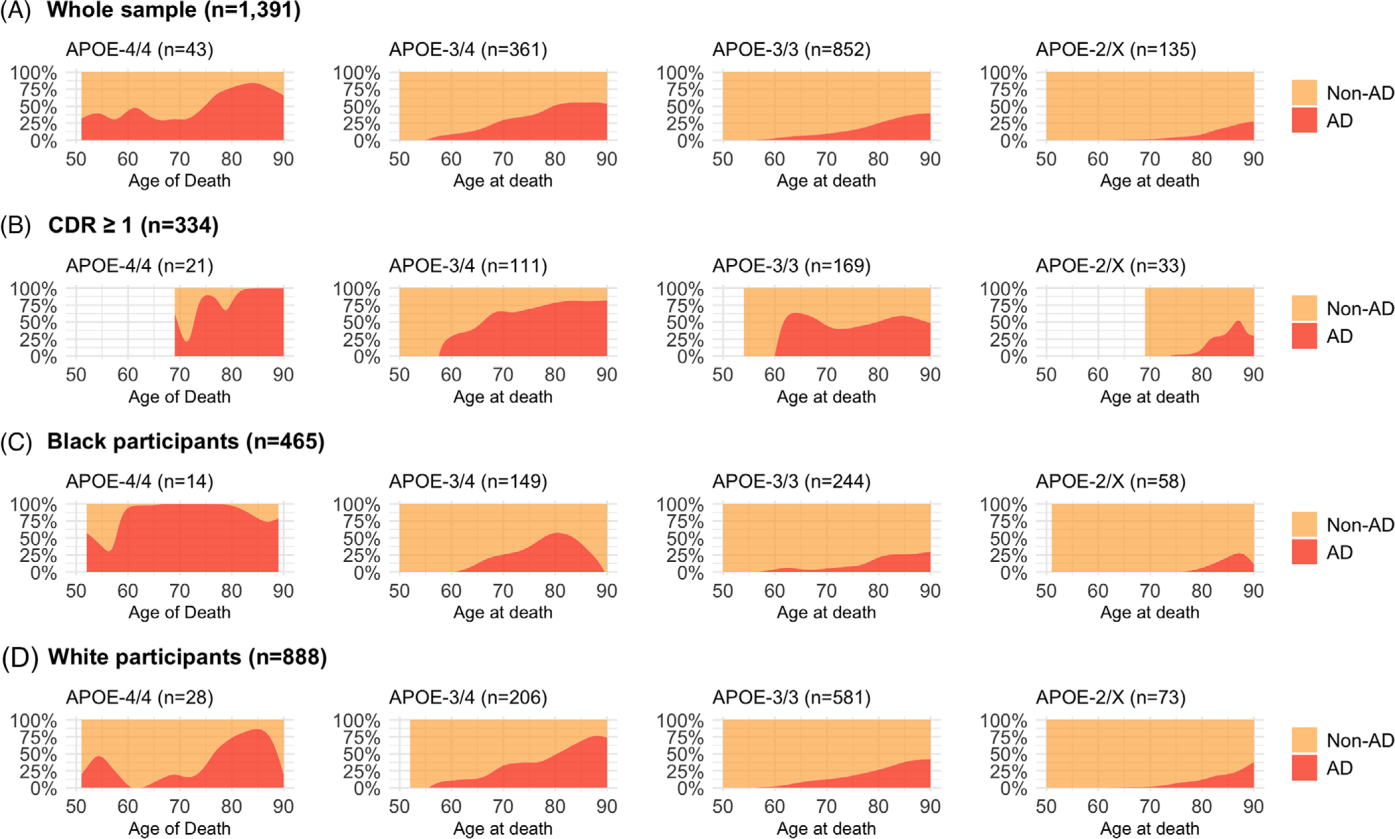
Distribution of Alzheimer's disease (AD) and non‐AD/normal neuropathological diagnoses by age of death and APOE genotypes in the whole sample (A) and stratified by Clinical Dementia Rating (CDR ≥ 1) and race (B and C). APOE, apolipoprotein E gene.

When we inspected the distribution of Braak staging across age groups, most APOE ε4 homozygotes showed higher or intermediate Braak stages compared to other APOE genotypes. By age 80, almost all APOE ε4 homozygotes showed higher or intermediate Braak stages (Figure 1A). The distribution of CERAD varied across age groups, with over 50% to 75% of APOE ε4 homozygotes having intermediate/high scores and around 75% in age groups older than 80 years old (Figure 1B). Regarding the Thal stages, intermediate and high scores were present in 50% to 75% of participants depending on the age groups (Figure 1B,C). However, the penetrance for APOE ε4/ε4 was not 100% for intermediate and high AD pathology in most age groups, despite most participants being older than 75 years and homozygotes for APOE ε4 having higher or intermediate Braak scores (Figure 2). The pattern remained consistent regardless of race. Similar patterns were noted for CERAD and Thal scores, with nearly all Black participants older than 60 years with APOE ε4/ε4 genotype showing higher CERAD/Thal scores compared to other genotypes (Figures 3 and S2). These patterns were also observed when the sample was stratified by CDR status, especially in those with cognitive impairment (CDR > 0) (Figures S3–S5). In plots exploring neuropathological diagnoses by APOE genotype and CDR status, we observed an APOE gene dose response for higher frequencies of AD pathology across groups with normal cognition to dementia (Figure S6). In participants with APOE ε4 homozygosis, the overall frequencies of AD pathological diagnosis were 86% among participants with dementia, 67% in those with questionable dementia, and 38% among participants with normal cognition (Figure S6). The frequency of AD pathology increased with age, especially in participants with normal cognition, such that by age 80, 100%, 60%, and 75% had intermediate or high Braak, CERAD, or Thal scores, respectively. Moreover, we presented the frequency for AD diagnosis and non‐AD/normal neuropathological evaluation for the whole sample, in participants with dementia (CDR ≥ 1), and stratified by race. The frequency of AD neuropathological diagnosis was not 100% in any age group for the whole sample. Still, it was 100% for those with dementia after 85 and in Black participants from 60 to almost 80 (Figure 4).

The mean age at death of the overall sample was 74.1 (95% CI = 72.0 to 76.2) and did not differ by APOE genotype (Table 2). However, the mean age at death in participants with AD pathology was higher in those with APOE ε2/εX (86.7, 95% CI = 83.6 to 89.8) and was progressively lower across APOE ε3/ε3, ε3/ε4, and ε4/ε4, with the lowest age at death among those with APOE ε4/ε4 (79.0, 95% CI = 74.8 to 83.2). The same pattern was noted in participants with preclinical AD pathology (CDR = 0, *n* = 115), while it was not seen in participants with questionable dementia (CDR = 0.5, *n* = 35) and dementia (CDR ≥ 1, *n* = 195) (Table 2). We also examined whether the age of death differed by race among APOE genotypes. Age of death decreased from APOE ε2/εX to APOE ε4/ε4 in White participants (*n* = 244), but the same pattern was not significant among Black participants, probably due to the small sample size (*n* = 92).

**TABLE 2 alz70927-tbl-0002:** Mean age at death and 95% confidence intervals according to apolipoprotein E genotypes in the overall sample and participants with Alzheimer`s disease pathology by cognitive status and race.

Variables	*n*	APOE ε2/εX	APOE ε3/ε3	APOE ε3/ε4	APOE ε4/ε4	*p* ^*^
Overall	1391	74.1 (72.0‐76.2)	75.1 (74.2‐76.0)	75.2 (74.0‐76.3)	77.2 (73.5‐81.0)	0.53
AD pathology	345	86.7 (83.6‐89.8)	84.8 (83.6‐86.0)	81.6 (80.1‐83.0)	79.0 (74.8‐83.2)	<0.001
CDR = 0	115	86.2 (82.5‐90.0)	84.0 (82.1‐85.9)	77.8 (75.1‐805)	70.1 (55.9‐84.2)	<0.001
CDR = 0.5	35	85.5 (65.6‐105.4)	85.0 (81.0‐89.0)	85.4 (78.6‐92.2)	69.4 (54.5‐84.4)	0.20
CDR ≥ 1	195	87.4 (84.0‐90.7)	85.2 (83.5‐86.9)	83.2 (81.7‐84.8)	82.7 (79.8‐85.7)	0.20
Black	92	84.7 (82.7‐86.8)	85.0 (81.5‐88.5)	80.7 (78.6‐82.9)	78.0 (69.9‐86.0)	0.09
White	244	87.3 (83.3‐91.4)	84.4 (83.1‐85.7)	81.8 (80.0‐83.7)	78.9 (74.1‐83.7)	0.01

*Note*: APOE ε2/ε4 was excluded from the analyses.

Abbreviations: AD, Alzheimer's disease; APOE, apolipoprotein E gene; CDR, Clinical Dementia Rating

* One‐way ANOVA test.

### 3.3 Association between APOE genotypes and AD pathology

Logistic regression models adjusted for age, sex, race, and education confirmed the associations of APOE genotypes with AD pathology and diagnosis (Table S1). APOE ε2/εX was related to lower odds of amyloid pathology (CERAD: OR = 0.45, 95% CI = 0.28 to 0.72, *p* = 0.001 and Thal: OR = 0.39, 95% CI = 0.25 to 0.58, *p* < 0.001) and AD diagnosis for the whole sample (OR = 0.38, 95% CI = 0.20 to 0.74, *p* = 0.005) and was also protective against neurofibrillary accumulation among participants with dementia (OR = 0.19, 95% CI = 0.09 to 040, *p* = 0.03). APOE ε3/ε4 and APOE ε4/ε4 were associated with higher odds of amyloid (CERAD‐ APOE ε3/ε4: OR = 3.75, 95% CI = 2.90 to 4.83, *p* < 0.001; APOE ε4/ε4: OR = 7.55, 95% CI = 4.16 to 13.69, *p* < 0.001) and tau accumulation (APOE ε3/ε4: OR = 1.78, 95% CI = 1.42 to 2.23, *p* < 0.001; APOE ε4/ε4: OR = 3.99, 95% CI = 2.25 to 7.08, *p* < 0.001), as well as AD neuropathological diagnosis (APOE ε3/ε4: OR = 3.07, 95% CI = 2.24 to 4.21, *p* < 0.001; APOE ε4/ε4: OR = 6.38, 95% CI = 3.11 to 13.08, *p* < 0.001) (Table S1). Although APOE ε4/ε4 seemed to exhibit higher odds of AD pathology than APOE ε3/ε4 carriers, most confidence intervals for APOE ε3/ε4 and APOE ε4/ε4 genotypes overlapped, probably due to the small number of participants with the APOE ε4/ε4 genotype (Table S1). Similar associations between APOE genotypes and AD pathology were found among Black and White participants (*p* for the interaction of race with CERAD = 0.36; Thal *p* = 0.20; Braak *p* = 0.41; and AD diagnosis *p* = 0.37).

## 4 DISCUSSION

In this diverse population‐based study, APOE ε4/ε4 was associated with a higher burden of AD pathology and neuropathological diagnosis. While APOE ε4 homozygotes had the highest frequencies of intermediate to high Braak, CERAD, and Thal stages, particularly in older age groups, full penetrance was not observed. Moreover, a gene dose response was evident across informant‐based cognitive status, with the frequency of neuropathological AD diagnosis in APOE ε4 homozygotes ranging from 38% in cognitively normal individuals to 86% in those with dementia. APOE ε2/εX was related to lower odds of AD pathology and diagnosis. Additionally, the age at death among individuals with AD pathology was inversely related to APOE gene dosage, with APOE ε4/ε4 carriers showing the youngest age at death and APOE ε2/εX the oldest age. Importantly, the association between APOE and AD pathology was similar across Black and White participants.

Several studies have suggested that the dose response of APOE alleles on the risk for a clinical AD dementia diagnosis varies by race.^12^, ^25^, ^26^ In a large study with more than 68,000 participants, the AD risk for APOE ε4 decreased following East Asian, White, Black, and Hispanic individuals.^12^ However, most of these studies relied on the clinical diagnosis without using AD‐related biomarkers,^12^, ^26^ which may have led to misclassification, particularly among older individuals where mixed pathologies are frequent.^27^ A study using amyloid positron emission tomography showed that APOE ε4 and APOE ε2 alleles had stronger associations with amyloid burden in White individuals, followed by African Americans, and lowest in Asian populations.^25^ Autopsy studies, which allow for a definitive AD diagnosis, can contribute to understanding whether the associations of APOE genotypes and AD pathology differ by race. APOE ε4 carriers had an elevated risk of neuropathologically characterized AD diagnosis, while APOE ε2/ε2 had lower odds.^15^ Similarly, high rates of AD pathology were reported among APOE ε4 homozygotes.^8^ Nevertheless, these studies used convenience samples, enriched for White participants and dementia diagnoses, and therefore may overestimate the penetrance of APOE ε4. Indeed, 99% and 88% of the brains in those studies had a diagnosis of AD dementia *ante mortem* compared to 49% in our study.^8^, ^15^ The ages at death were also significantly different: 75 years in our study versus 83 and 82 years in Fortea's and Reiman's studies, respectively.

Although APOE ε4/ε4 was strongly associated with AD pathology in our sample, full penetrance was not observed across all ages, even among individuals with dementia. A proportion of APOE ε4 carriers without dementia still harbor AD pathology, supporting the concept that APOE ε4 confers increased vulnerability long before the onset of overt clinical symptoms. Conversely, the observation that some APOE ε4 carriers remain cognitively intact despite AD pathology suggests the presence of resilience factors, such as cognitive reserve, lifestyle influences, or protective genetic variants, that warrant further study. This discrepancy from previous studies may be due to differences in study design, racial and socioeconomic diversity, and the methodological advantage of a population‐based autopsy study.^8^ The frequency of APOE ε4/ε4 was 12.6% in the Reiman et al. study and 8.3% in the Fortea et al. study, while it was 3.1% in our sample. Reiman´s study used the Alzheimer's Disease Genetics Consortium (ADGC) dataset, while Fortea's study used the NACC sample; both datasets are enriched with individuals with advanced dementia and have few participants with normal cognition. Nonetheless, Fortea's study also had clinical cohorts with 57% cognitively unimpaired individuals, 22% with MCI, and 21% with AD dementia. Although the biological penetrance of amyloid and tau biomarkers with age in those studies was similar to our findings, the frequency of intermediate and high CERAD scores in our study is lower, and full penetrance of APOE4/4 for AD pathology with age was not observed as defined by the ADNC criteria.

The frequency of APOE ε4/ε4 in our study is in line with a Brazilian population‐based study,^24^ which found a frequency of 2.2% for APOE ε4/ε4, and with a 1.1% frequency in the Genome Aggregation Database (gnomAD).^28^ Our study contributes novel insights into the role of APOE across racial groups, as we compared Black and White participants. Despite the smaller number of APOE ε4/ε4 and APOE ε2/εX carriers in racial subgroups, we found no significant differences in the strength of association between APOE genotypes and AD pathology by race. This suggests that, within the limitations of our sample size, the impact of APOE on AD pathology may be consistent across different races.

The biological mechanisms underlying the dose response of APOE on AD pathology involve Aβ aggregation and clearance processes.^6^ APOE ε4 carriers exhibit higher levels of soluble Aβ oligomers, with in vitro studies demonstrating that APOE ε4 promotes Aβ oligomerization, whereas APOE ε2 and APOE ε3 inhibit the conversion of protofibrils into fibrils.^29^ APOE isoforms also differ markedly in their ability to mediate Aβ degradation and clearance.^6^ APOE ε4 is less effective at facilitating the removal of soluble Aβ from the brain interstitial fluid, and its uptake and subsequent degradation by astrocytes, microglia, and neurons are impaired compared to APOE ε2 and APOE ε3.^30^ Moreover, at the blood–brain barrier (BBB), APOE ε2 and APOE ε3 promote Aβ clearance via both the low‐density lipoprotein receptor‐related protein 1 (LRP1) and the very low‐density lipoprotein receptor (VLDLR), whereas APOE ε4 preferentially uses VLDLR alone, resulting in reduced efficiency of Aβ elimination.^31^ Additional clearance pathways mediated by LDL receptors in astrocytes and neurons are less effective in the presence of APOE ε4.^32^ Furthermore, APOE ε4 has been shown to impair Aβ transport across the BBB.^32^ The impact of APOE ε4 on tau pathology appears to be largely indirect, though the acceleration of tau deposition by the Aβ deposition.^33^ These findings highlight distinct mechanisms through which APOE modulates AD pathogenesis, with APOE ε4 enhancing and APOE ε2 attenuating processes related to Aβ.^6^

While APOE isoforms seem to have robust biological effects on AD pathology,^6^ we did not observe racial differences in their associations with AD neuropathology, suggesting that the molecular pathways through which APOE influences AD are largely conserved across populations. Alternatively, racial differences in APOE effects reported in other studies may arise from factors not related to APOE biology, such as ancestry‐related genetic variation or unequal social conditions.^25^ In Brazil, where racial classification is based on skin color and the population is highly admixed, biological differences by race may be attenuated compared with studies in the US.^17^, ^24^ Moreover, our analyses were adjusted for socioeconomic variables, which may have minimized differences between racial groups. Taken together, these findings suggest that while APOE shapes AD pathology through shared biological mechanisms, racial disparities in dementia expression may depend more on environmental and social modifiers than on differences in molecular biology.^9^

This study has several strengths. First, it is one of the largest autopsy‐based investigations of the association between APOE genotypes and AD pathology conducted in a diverse population. Our inclusion of participants from different racial and socioeconomic backgrounds addresses a critical gap in the literature, which has been predominantly based on White populations from high‐income countries.^8^, ^15^ In Brazil, however, racial classification is based primarily on self‐identification, with high levels of admixture,^24^ making comparisons with US racial categories challenging.^17^ Structural inequalities are similar in Brazil and the US, with Black Brazilians experiencing greater socioeconomic disadvantage, which itself may modify the relationship[Table alz70927-tbl-0002] between APOE genotype and dementia.^17^ Although our models were adjusted for socioeconomic factors, the lack of genetic ancestry data remains a limitation. Including such data in future studies would help distinguish ancestry‐related genetic effects from social and environmental influences and provide a more nuanced understanding of how APOE and ancestry interact to shape AD pathology in admixed populations. Another key strength of the study, beyond the diversity of the sample, is that it was nearly population‐based, as evidenced by APOE haplotype frequencies closely mirroring those of the general Brazilian population; however, the dementia penetrance may have been underestimated due to inclusion criteria favoring individuals who died of unknown causes. Additionally, we used rigorous and standardized neuropathological assessments combined with genetic data, providing a robust characterization of AD pathology across APOE genotypes. Nonetheless, some limitations should be acknowledged. Although the overall sample size was large, the number of participants with APOE ε4/ε4 and APOE ε2/εX genotypes was relatively small, limiting our ability to perform more detailed analyses stratified by race for these specific genotypes and increasing the chances of type 2 error. Cognitive status was determined after death through structured informant interviews; although a previous validation study demonstrated good accuracy of this method compared to direct patient assessment,^16^ some degree of misclassification remains possible. Moreover, the absence of clinical follow‐up before death precluded the evaluation of longitudinal cognitive trajectories. Thus, we were unable to directly assess the temporal sequence linking APOE genotype, pathology accumulation, and conversion to clinical dementia. Future research should include longitudinal clinical–pathological studies to address this gap. In addition, brains with major cerebrovascular lesions were excluded because of the need for brain retention by the pathologist in charge of the autopsy exam to determine the cause of death. This exclusion could lead to selection bias, which could overestimate the association between APOE and AD pathology by reducing the representation of individuals with mixed or predominantly vascular dementia. Finally, this was an observational study, which limits our ability to draw causal inferences between APOE genotype and AD pathology.

Our findings reinforce the role of APOE genotypes in AD pathogenesis and underscore the utility of autopsy studies in diverse populations to refine our understanding of genetic risk and disease expression. Other diverse autopsy studies are necessary to validate and extend the knowledge about whether the association between APOE and AD pathology differs by race.

## CONSENT STATEMENT

A close family member authorized the brain donation and signed an informed consent document for study participation.

## CONFLICT OF INTEREST STATEMENT

J.F. reported serving on the advisory boards or adjudication committees or receiving speaker honoraria from AC Immune, Adamed, Alzheon, Biogen, Eisai, Esteve, Fujirebio, Ionis, Laboratorios Carnot, Life Molecular Imaging, Lilly, Lundbeck, Novo Nordisk, Perha, Roche, Zambón, Spanish Neurological Society, T21 Research Society, Lumind Foundation, Jérôme‐Lejeune Foundation, Alzheimer's Association, National Institutes of Health USA, and Instituto de Salud Carlos III. J.F. reports holding a patent for markers of synaptopathy in neurodegenerative disease (licensed to ADx, EPI8382175.0). A.C.P. has patents licensed to Neurobiopharma, LLC (unrelated to this work), serves on the scientific advisory board of Sinaptica Therapeutics and Tau Biosciences, and has served as a consultant to Eisai and Quanterix. Other authors have nothing to disclose. Author disclosures are available in the Supporting Information.

## Supporting information



Supporting Information

Supporting Information
