# Supplementary material for: Apolipoprotein E and Alzheimer's disease pathology in a diverse autopsy study
Source: Alzheimers Dement. 2025 Nov 21;21(11):e70927. doi: 10.1002/alz.70927 (PMC12635860; doi:10.1002/alz.70927)
Supplement: Supplementary file 1 — Supporting Information [file ALZ-21-e70927-s001.docx]

**Supplementary Material**

**Supplementary Figures**

**Supplementary Figure 1**. Flowchart of the study participants.

**Supplementary Figure 2.**  Distribution of Thal stages by age of death and APOE genotypes in the whole sample (a) and stratified by race (b, c). APOE: apolipoprotein E gene.

**Supplementary Figure 3**. Distribution of Braak stages by age of death and APOE genotypes. Panel A shows the distribution in the whole sample, Panel B shows the distribution stratified in CDR=0 (normal cognition), and Panel C shows the distribution in CDR>0 (cognitive impairment). CDR: Clinical Dementia Rating; APOE: apolipoprotein E gene.

**Supplementary Figure 4.** Distribution of CERAD score by age of death and APOE genotypes. Panel A shows the distribution in the whole sample. Panel B shows the distribution stratified in CDR=0 (normal cognition), and Panel C shows the distribution in CDR>0 (cognitive impairment). CDR: Clinical Dementia Rating; APOE: apolipoprotein E gene.

**Supplementary Figure 5.**  Distribution of Thal stages by age of death and APOE genotypes. Panel A shows the distribution in the whole sample. Panel B shows the distribution stratified in CDR=0 (normal cognition), and Panel C shows the distribution in CDR>0 (cognitive impairment). CDR: Clinical Dementia Rating; APOE: apolipoprotein E gene.

**Supplementary Figure 6.** Neuropathological diagnosis by APOE genotypes in participants with CDR=0, CDR=0.5, and CDR≥ 1 (dementia). AD: Alzheimer's disease; CDR: Clinical Dementia Rating; APOE: apolipoprotein E gene.

**Supplementary Table**

**Supplementary Figures**


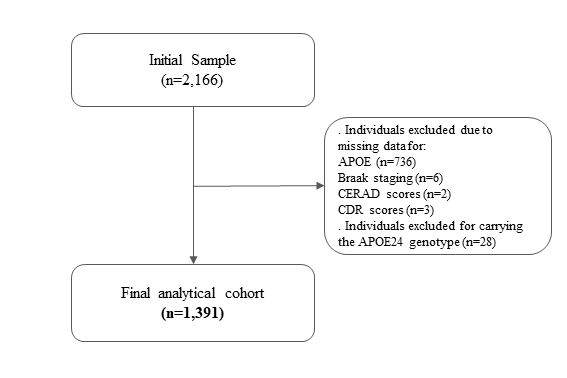


**Supplementary Figure 1**. Flowchart of the study participants.


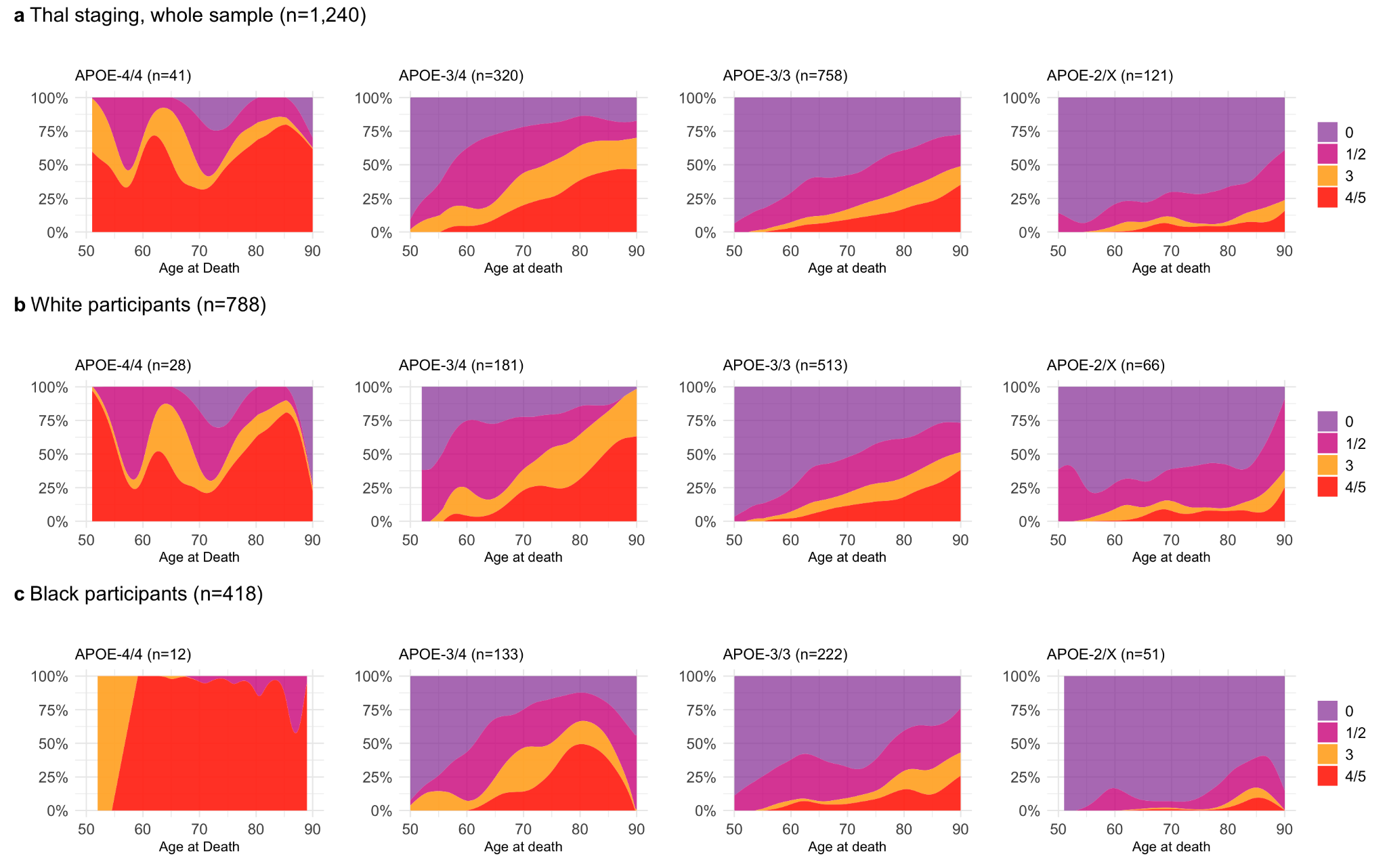


**Supplementary Figure 2.**  Distribution of Thal stages by age of death and APOE genotypes in the whole sample (a) and stratified by race (b, c). APOE: apolipoprotein E gene.


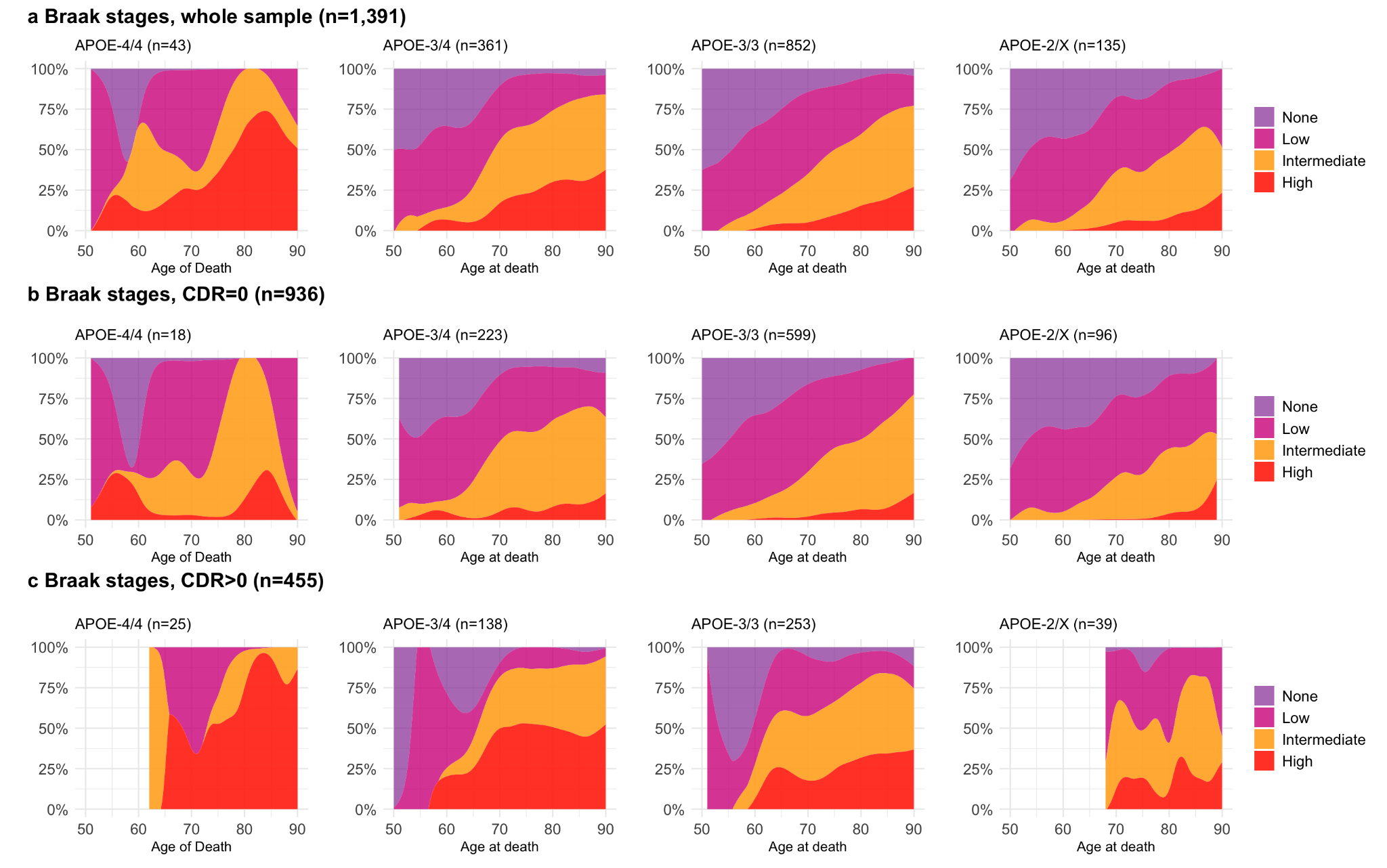


**Supplementary Figure 3**. Distribution of Braak stages by age of death and APOE genotypes. Panel A shows the distribution in the whole sample, Panel B shows the distribution stratified in CDR=0 (normal cognition), and Panel C shows the distribution in CDR>0 (cognitive impairment). CDR: Clinical Dementia Rating; APOE: apolipoprotein E gene.


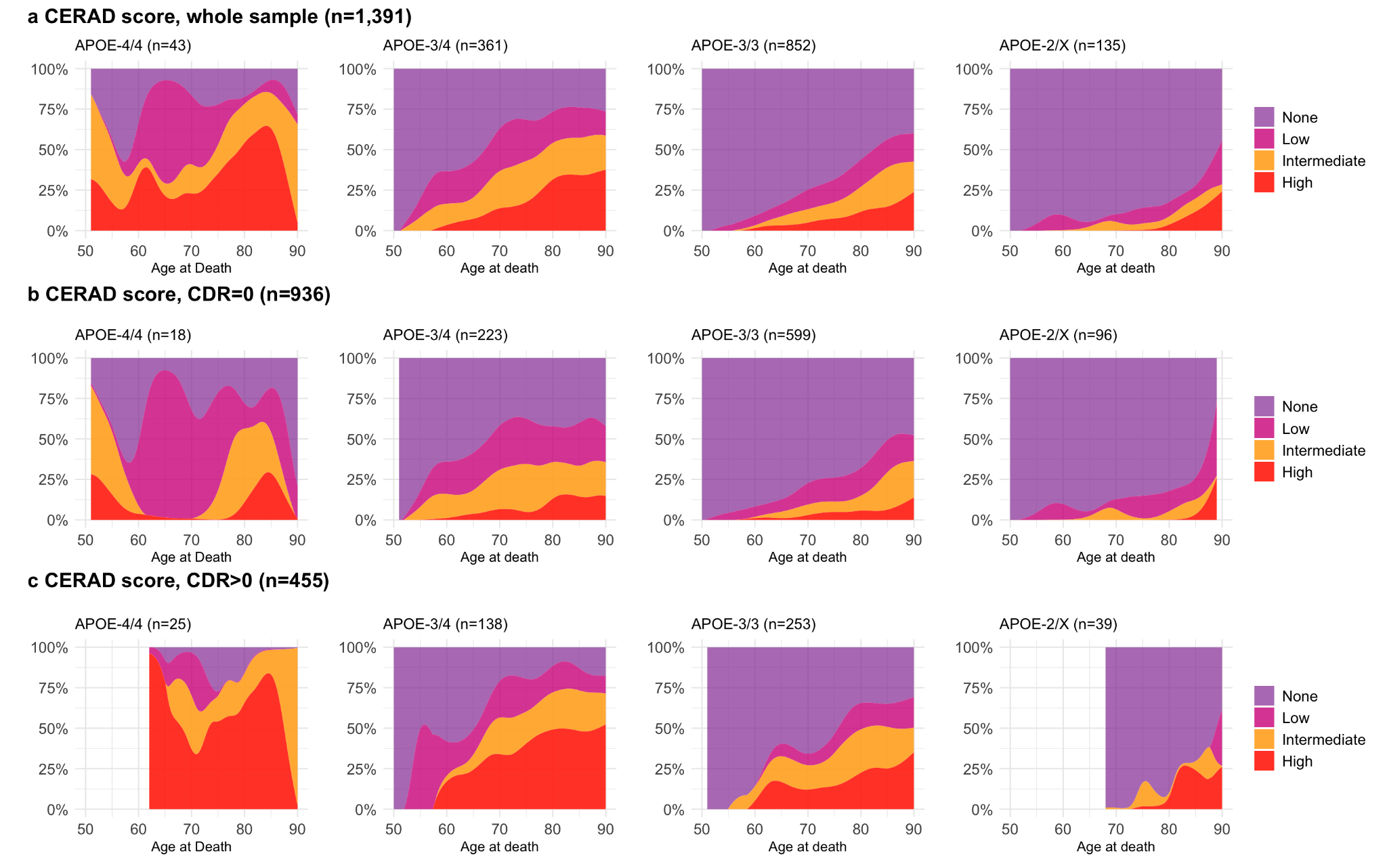


**Supplementary Figure 4.** Distribution of CERAD score by age of death and APOE genotypes. Panel A shows the distribution in the whole sample. Panel B shows the distribution stratified in CDR=0 (normal cognition), and Panel C shows the distribution in CDR>0 (cognitive impairment). CDR: Clinical Dementia Rating; APOE: apolipoprotein E gene.


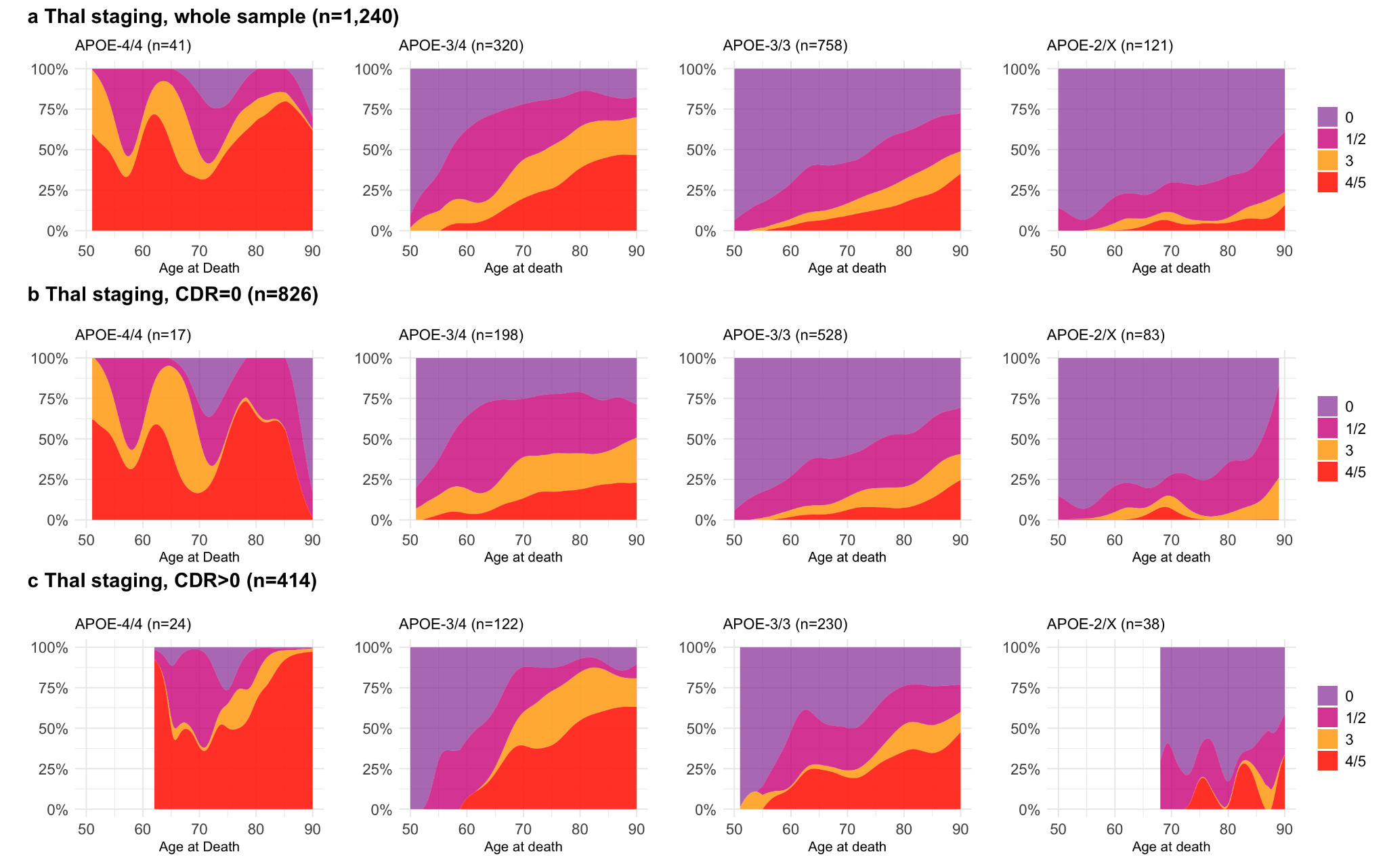


**Supplementary Figure 5.**  Distribution of Thal stages by age of death and APOE genotypes. Panel A shows the distribution in the whole sample. Panel B shows the distribution stratified in CDR=0 (normal cognition), and Panel C shows the distribution in CDR>0 (cognitive impairment). CDR: Clinical Dementia Rating; APOE: apolipoprotein E gene.


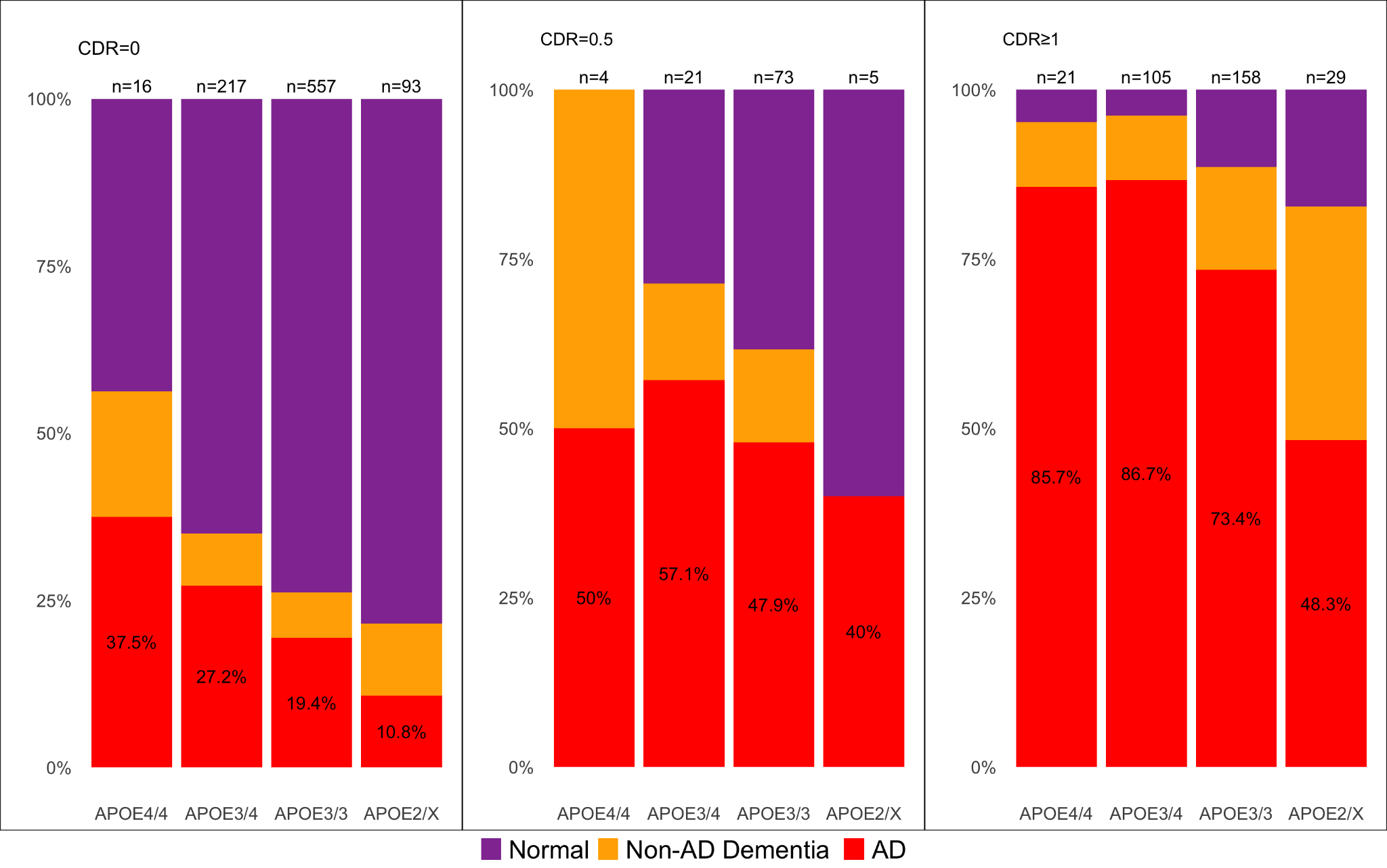


**Supplementary Figure 6.** Neuropathological diagnosis by APOE genotypes in participants with CDR=0, CDR=0.5, and CDR≥ 1 (dementia). AD: Alzheimer's disease; CDR: Clinical Dementia Rating; APOE: apolipoprotein E gene.

**Supplementary Table**

**Supplementary Table 1.** Association of APOE 3/4 and APOE 4/4 haplotypes with Alzheimer's disease neuropathological changes and diagnosis* (n=1,391).

|  | APOE2/X |  | APOE3/4 |  | APOE4/4 |  |
| --- | --- | --- | --- | --- | --- | --- |
|  | **OR (95% CI)** | **p** | **OR (95% CI)** | **p** | **OR (95% CI)** | **p** |
| Whole sample | **N=135** |  | **N=361** |  | **N=43** |  |
| CERAD^├^ | 0.45 (0.28-0.72) | 0.001 | 3.75 (2.90-4.83) | <0.001 | 7.55 (4.16-13.69) | <0.001 |
| Thal^├^ | 0.39 (0.25-0.58) | <0.001 | 3.21 (2.51-4.10) | <0.001 | 8.58 (4.67-15.75) | <0.001 |
| Braak^├^ | 0.72 (0.52-1.01) | 0.06 | 1.78 (1.42-2.23) | <0.001 | 3.99 (2.25-7.08) | <0.001 |
| AD diagnosis^¶^ | 0.38 (0.20-0.74) | 0.005 | 3.07 (2.24-4.21) | <0.001 | 6.38 (3.11-13.08) | <0.001 |
| Dementia (CDR ≥1) | **N=33** |  | **N=111** |  | **N=21** |  |
| CERAD^├^ | 0.23 (0.10-0.50) | <0.001 | 3.17 (1.98-5.06) | <0.001 | 3.86 (1.61-9.24) | 0.002 |
| Thal^├^ | 0.19 (0.09-0.40) | <0.001 | 2.09 (1.30-3.33) | 0.002 | 5.55 (2.30-13.39) | <0.001 |
| Braak^├^ | 0.50 (0.27-0.95) | 0.03 | 2.05 (1.32-3.19) | 0.001 | 3.33 (1.42-7.80) | 0.005 |
| AD diagnosis^¶^ | 0.19 (0.07-0.49) | 0.001 | 3.07 (1.75-5.38) | <0.001 | 4.72 (1.45-15.37) | 0.01 |
| Black (n=465) | **N=58** |  | **N=149** |  | **N=14** |  |
| CERAD^├^ | 0.35 (0.15-0.85) | 0.02 | 3.82 (2.44-5.98) | <0.001 | 12.50 (4.04-38.70) | <0.001 |
| Thal^├^ | 0.24 (0.11-0.51) | <0.001 | 2.93 (1.95-4.42) | <0.001 | 14.00 (4.21-46.59) | <0.001 |
| Braak^├^ | 0.58 (0.35-0.96) | 0.04 | 1.47 (1.01-2.12) | 0.04 | 3.40 (1.20-9.67) | 0.02 |
| AD diagnosis^¶^ | 0.34 (0.10-1.19) | 0.09 | 2.98 (1.71-5.21) | <0.001 | 16.7 (4.11-67.84) | <0.001 |
| White (n=888) | **N=73** |  | **N=206** |  | **N=28** |  |
| CERAD^├^ | 0.54 (0.31-0.95) | 0.03 | 3.65 (2.66-5.01) | <0.001 | 6.28 (3.04-12.98) | <0.001 |
| Thal^├^ | 0.52 (0.31-0.88) | 0.01 | 3.35 (2.45-4.57) | <0.001 | 6.83 (3.37-13.83) | <0.001 |
| Braak^├^ | 0.82 (0.53-1.28) | 0.39 | 2.06 (1.54-2.75) | <0.001 | 4.19 (2.10-8.36) | <0.001 |
| AD diagnosis^¶^ | 0.42 (0.19-0.93) | 0.03 | 3.20 (2.16-4.73) | <0.001 | 4.19 (1.76-9.97) | 0.001 |

*Alzheimer's disease (AD) neuropathological diagnosis was defined as a Braak staging ≥ 3 and a Consortium to Establish a Registry for Alzheimer’s Disease (CERAD) score ≥ 2.

^├^Ordinal logistic regression model adjusted for age, sex, race, and education

^¶^Binary logistic regression model adjusted for age, sex, race, and education.

OR: odds ratio. CI: confidence interval

Reference: APOE3/3 (whole sample: n=852; dementia: n=169; Black participants: n=244; White participants: n=581)

APOE2/4 was excluded from the analyses
